# Supplementary material for: Predicted mechanistic impacts of human protein missense variants
Source: bioRxiv. 2024 May 29:2024.05.29.596373. Preprint. [Version 1] doi: 10.1101/2024.05.29.596373 (PMC11160786; doi:10.1101/2024.05.29.596373)
Supplement: Supplement 1 [file NIHPP2024.05.29.596373v1-supplement-1.pdf]

bioRxiv preprint doi: https://doi.org/10.1101/2024.05.29.596373; this version posted May 29, 2024. The copyright holder for this preprint (which was not certified by peer review) is the author/funder, who has granted bioRxiv a license to display the preprint in perpetuity. It is made available under aCC-BY 4.0 International license.

Supplementary Figures

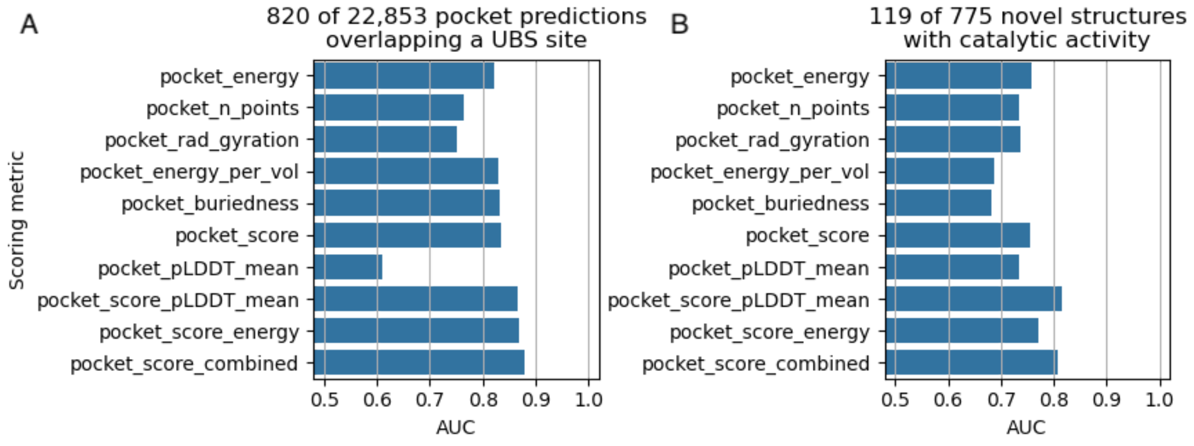

Fig S1 - Recall of known binding sites (top) and enzymatic activity (bottom) using different pocket prediction scoring metrics
pocket_energy, pocket_n_points, pocket_rad_gyration, pocket_energy_per_vol, pocket_buriedness and (AutoSite) pocket_score are defined as in (Ravindranath & Sanner, 2016); pocket_pLDDT_mean is the mean pLDDT values of pocket-associated residues (within 4.5A of the pocket). pocket_score_pLDDT_mean, pocket_score_energy and pocket_score_combined are modifications of the AutoSite score defined as follows:

score = n_points * buriedness**2 / radius_gyration
score_pLDDT_mean = n_points * buriedness**2 * pLDDT_mean**2 / radius_gyration
score_energy = energy * buriedness**2 / radius_gyration
score_combined = energy * buriedness**2 * pLDDT_mean**2 / radius_gyration

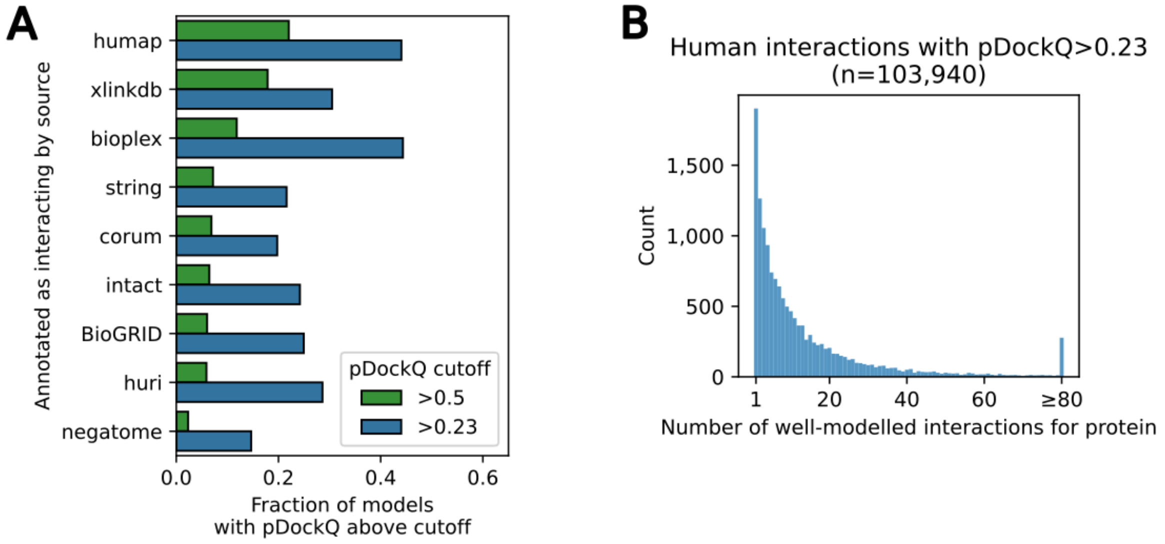

Fig S2 - Predicted models per interaction type and binned per protein.
A. pDockQ confidence scores for interactions reported in different databases. A higher fraction of confident models (pDockQ>0.5) was observed for interactions derived by affinity-purification (humap, bioplex) and cross-linking data (xlinkdb) than for yeast-two-hybrid (huri) or general compilations of data (BioGRID, intAct).
B. Number of models with pDockQ>0.23 per protein. The distribution of the number of models per protein follows a heavy tail distribution that is similar to the distribution of the number of experimentally determined protein-protein interactions per protein. The majority of proteins having few (<15) interaction models with a long tail of proteins having a large number of models.

bioRxiv preprint doi: https://doi.org/10.1101/2024.05.29.596373; this version posted May 29, 2024. The copyright holder for this preprint (which was not certified by peer review) is the author/funder, who has granted bioRxiv a license to display the preprint in perpetuity. It is made available under aCC-BY 4.0 International license.

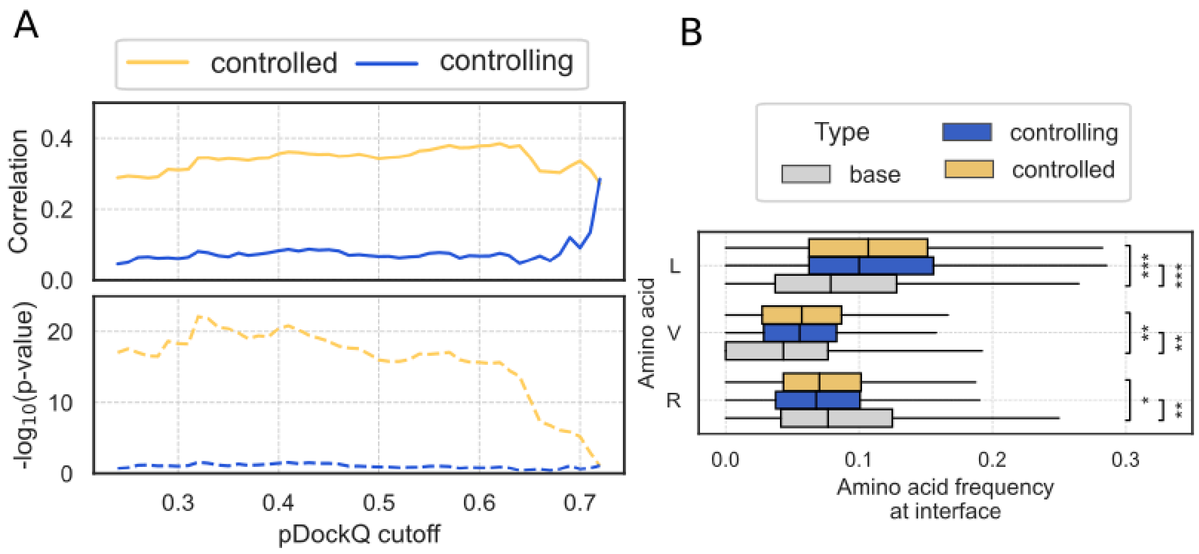

Fig S3 - Relation protein surface properties and interaction mediated control of protein abundance.

A. Correlation (upper plot) and significance of correlation (lower plot) between the strength of attenuation (CNV Beta) and the fraction of protein residues at the interaction interface in the controlled subunit (yellow) and in the controlling subunit (blue) for different pDockQ cutoffs.

B. Enrichment of leucine and valine residues and depletion of arginine residues at the interfaces where there is a strong predicted control of protein levels by protein-protein interactions.
